# Chytrids alleviate the harmful effect of heat and cyanobacteria diet on *Daphnia* via PUFA-upgrading

**DOI:** 10.1093/plankt/fbad012

**Published:** 2023-04-21

**Authors:** András Abonyi, Matthias Pilecky, Serena Rasconi, Robert Ptacnik, Martin J Kainz

**Affiliations:** WasserCluster Lunz – Biologische Station GmbH, Dr. Carl Kupelwieser Promenade 5, A-3293 Lunz am See, Austria; Institute of Aquatic Ecology, Centre for Ecological Research, Karolina út 29, H-1113 Budapest, Hungary; WasserCluster Lunz – Biologische Station GmbH, Dr. Carl Kupelwieser Promenade 5, A-3293 Lunz am See, Austria; Donau-Universität Krems, Dr. Karl Dorrek Straße 30, A-3500 Krems, Austria; WasserCluster Lunz – Biologische Station GmbH, Dr. Carl Kupelwieser Promenade 5, A-3293 Lunz am See, Austria; Université Savoie Mont Blanc, INRAE, UMR CARRTEL, 75 bis avenue de Corzent, Thonon les Bains cedex F-74200, France; WasserCluster Lunz – Biologische Station GmbH, Dr. Carl Kupelwieser Promenade 5, A-3293 Lunz am See, Austria; WasserCluster Lunz – Biologische Station GmbH, Dr. Carl Kupelwieser Promenade 5, A-3293 Lunz am See, Austria; Donau-Universität Krems, Dr. Karl Dorrek Straße 30, A-3500 Krems, Austria

**Keywords:** climate change, compound-specific FA, δ13C, ecosystem functioning, mycoloop

## Abstract

Chytrid fungal parasites increase herbivory and dietary access to essential molecules, such as polyunsaturated fatty acids (PUFA), at the phytoplankton–zooplankton interface. Warming enhances cyanobacteria blooms and decreases algae-derived PUFA for zooplankton. Whether chytrids could support zooplankton with PUFA under global warming scenarios remains unknown. We tested the combined effect of water temperature (ambient: 18°C, heat: +6°C) and the presence of chytrids with *Daphnia magna* as the consumer, and *Planktothrix rubescens* as the main diet. We hypothesized that chytrids would support *Daphnia* fitness with PUFA, irrespective of water temperature. Heating was detrimental to the fitness of *Daphnia* when feeding solely on the *Planktothrix* diet. Chytrid-infected *Planktothrix* diet alleviated the negative impact of heat and could support *Daphnia* survival, somatic growth and reproduction. Carbon stable isotopes of fatty acids highlighted a ~3x more efficient n-3 than n-6 PUFA conversion by *Daphnia* feeding on the chytrid-infected diet, irrespective of temperature. The chytrid diet significantly increased eicosapentaenoic acid (EPA; 20:5n-3) and arachidonic acid (ARA; 20:4n-6) retention in *Daphnia*. The EPA retention remained unaffected, while ARA retention increased in response to heat. We conclude that chytrids support pelagic ecosystem functioning under cyanobacteria blooms and global warming via chytrids-conveyed PUFA toward higher trophic levels.

## INTRODUCTION

Recent evidence suggests that chytrid fungal parasites perform vital functions at the phytoplankton–zooplankton interface. Chytrids enhance herbivory by fragmenting poorly edible algal/cyanobacteria hosts (Frenken *et al.*, 2020). They also improve the dietary supply of polyunsaturated fatty acids (PUFA; Gerphagnon *et al.*, 2019; Rasconi *et al.*, 2020) and sterols (Kagami *et al.*, 2007; Gerphagnon *et al.*, 2019), as essential molecules, for zooplankton. Chytrids are ubiquitous, invading various phytoplankton hosts (Gleason *et al.*, 2015) and producing edible (~2–5 μm) free-swimming zoospores for subsequent consumers, such as zooplankton. Chytrids thus provide an alternative dietary energy pathway between phytoplankton and zooplankton, called the “mycoloop” (Kagami *et al.*, 2014). Chytrids upgrade dietary carbon from the algal/cyanobacteria hosts in terms of omega-3 PUFA and sterols (Gerphagnon *et al.*, 2019; Rasconi *et al.*, 2020), which positively affects the fitness of *Daphnia* as a subsequent consumer (Abonyi *et al.*, 2023). Whether and how the mycoloop can support zooplankton with enhanced PUFA availability under global warming (IPCC, 2021) remains largely unexplored.

The efficient transfer of dietary energy and essential molecules across the phytoplankton–zooplankton trophic interface is crucial for consumers at higher trophic levels. The energy transfer can be constrained quantitatively and qualitatively. A quantitative limitation may occur in response to reduced primary production, e.g. during oligotrophication (Jeppesen *et al.*, 2002; Jochimsen *et al.*, 2013). Alternatively, increased primary production in response to eutrophication and global warming may result in the more frequent proliferation of harmful phytoplankton (Paerl and Huisman, 2008; O’Neil *et al.*, 2012). Bloom-forming cyanobacteria are typically filamentous or colonial, which hinders zooplankton grazing and creates an energetic bottleneck between pelagic primary producers and herbivorous consumers (Havens, 2008). Qualitative limitation of energy transfer may also be linked to phytoplankton blooms. Cyanobacteria especially lack essential compounds for zooplankton, such as PUFA and sterols, so their proliferation truncates the trophic transfer of highly required dietary compounds.

Zooplankton cannot synthesize PUFA *de novo* (Demott and Müller-Navarr, 1997; Gulati and Demott, 1997). The omega-3 PUFA eicosapentaenoic acid (EPA, 20:5n-3) and the omega-6 PUFA arachidonic acid (ARA, 20:4n-6) are the two conditionally indispensable molecules for zooplankton growth and reproduction (see, e.g. Müller-Navarra *et al.*, 2000; Sikora *et al.*, 2016; Ilić *et al.*, 2021). They can be obtained directly from the diet or converted from their respective precursors, linoleic acid (LIN, 18:2n-6) and alpha-linolenic acid (ALA, 18:3n-3), respectively (Pilecky *et al.*, 2021). A key source of PUFA for herbivorous zooplankton in aquatic food webs is phytoplankton, which vary widely in PUFA composition (Taipale *et al.*, 2013). Diatoms are rich in EPA; cryptophytes contain EPA and docosahexaenoic acid (DHA, 22:6n-3); thus, both groups are considered high-quality diets for zooplankton. Green algae contain mostly LIN and ALA and are referred to as intermediate diet quality. Cyanobacteria generally lack long-chain PUFA and sterols (but see the gamma-linoleic acid, GLA, 18:3n-6, content of *Microcystis* sp.; Strandberg *et al.*, 2020) and are thus low-quality diets for zooplankton (Martin-Creuzburg *et al.*, 2009).

Hardly palatable cyanobacteria of poor diet quality are deleterious for zooplankton (Bednarska *et al.*, 2014), representing a worst-case scenario in the trophic transfer of energy and essential dietary molecules. Recent evidence suggests that chytrid fungal parasites can buffer this effect by enhancing trophic transfer from cyanobacteria to zooplankton during cyanobacteria blooms (Agha *et al.*, 2016; Abonyi *et al.*, 2023). In previous studies, the presence of chytrids enhanced the somatic growth and reproduction of cladocerans (Kagami *et al.*, 2007; Agha *et al.*, 2016), copepods (Kagami *et al.*, 2011) and rotifers (Frenken *et al.*, 2018) when growing on a poor quality diet. Mechanisms potentially underlying the positive dietary effects of chytrids are (1) enhanced herbivory by fragmenting otherwise inedible cyanobacteria (Agha *et al.*, 2016; Frenken *et al.*, 2020), and (2) increased nutritional quality in terms of PUFA (Gerphagnon *et al.*, 2019; Taube *et al.*, 2019; Rasconi *et al.*, 2020). Chytrids can upgrade short-chain to long-chain PUFA from the host, i.e. convert the omega-3 PUFA ALA to stearidonic acid (SDA) and then further to long-chain PUFA, such as EPA (Rasconi *et al.*, 2020). Recent evidence suggests that SDA synthesized by chytrids is selectively retained by *Daphnia* with a concomitant increase in the fitness of the consumer (Abonyi *et al.*, 2023). How the mycoloop and the conveyed PUFA interact with heat remains a crucial issue, especially to understand better the combined effects of warming and increased occurrence of phytoplankton blooms.

Warming amplifies the deleterious effect of reduced diet intake on ectothermic organisms (Huey and Kingsolver, 2019). Hardly edible PUFA-deprived cyanobacteria blooms thus may help collapse the populations of herbivorous consumers, especially under water temperature increase. In light of ongoing global warming (IPCC, 2021) and the more frequent occurrence of cyanobacteria blooms in aquatic ecosystems, it is crucial to test the strength of the mycoloop in warmer conditions. If improved dietary PUFA provision via chytrids enhances *Daphnia* fitness on top of increased herbivory (Abonyi *et al.*, 2023), it may also be able to provide insurance against increasing water temperature.

Here we ask whether and how chytrids can support *Daphnia* fitness under water temperature increase and stress induced by the detrimental interaction of heat and hardly available dietary carbon and PUFA. Masclaux *et al.* (2009) showed a trade-off in *Daphnia* growth rate along with diet quality and water temperature. If a high-quality diet was available *ad libitum*, water temperature increase enhanced *Daphnia* growth, while reduced diet quality or quantity alleviated the positive temperature effect (Masclaux *et al.*, 2009). On the other hand, multiple observations showed that *Daphnia* growth rate decreased at water temperatures >20°C (Giebelhausen and Lampert 2001; Masclaux *et al.*, 2009), but it remained an essentially optimal condition for growth under non-limiting diet conditions (Müller *et al.*, 2018). *Daphnia* adjust FA content to the water temperature, decreasing PUFA toward higher temperatures (Zeis *et al.*, 2019). Dietary EPA limitation was also shown to be more critical at lower than higher water temperatures (Sperfeld and Wacker, 2012). The fitness of *Daphnia magna* is already constrained at 18°C when feeding on poor diet quality and hardly edible *Planktothrix rubescens* (Abonyi *et al.*, 2023). We thus performed a feeding experiment where *D. magna* was supplied with *Planktothrix rubescens* alone—a filamentous bloom-forming cyanobacterium—or with chytrid-infected *Planktothrix*. We combined the two alternative diets with two water temperature treatments, i.e. ambient (18°C) and heat (ambient +6°C). Applying a 24°C heat treatment enabled us to study the PUFA transfer via the mycoloop with water temperature increase without directly inducing *Daphnia* mortality by heat-mediated metabolic stress (Zeis *et al.*, 2019; Im *et al.*, 2020).

We hypothesized that a +6°C water temperature increase in combination with a filamentous cyanobacterium diet would be detrimental to *Daphnia* fitness, also mediated by PUFA-deprived conditions. We expected that diets including chytrids would support *Daphnia* fitness due to increased PUFA transfer even under increased water temperature. We examined survival, somatic growth and reproduction of *Daphnia* as well as PUFA accrual of *Daphnia* among the treatments. In a ^13^C-labeling approach, we also aimed at identifying the trophic carbon pathways via which physiologically functional PUFA were transferred to and converted by *Daphnia*.

## MATERIAL AND METHODS

### Experimental cultures

The filamentous cyanobacterium *Planktothrix rubescens* (strain NIVA-CYA97/1) was cultured and maintained alone, and together with its specific chytrid fungal parasite (strain Chy-Lys2009) on WC Medium (Guillard and Lorenzen, 1972). We maintained a high chytrid prevalence rate (>50%) in the chytrid-infected *Planktothrix* culture by 1/3 V/V% medium- and 1/3 V/V% dense uninfected *Planktothrix* culture-exchange once a week (Abonyi *et al.*, 2023). We used the chytrid-infected *Planktothrix* culture as a diet without separating chytrid zoospores and *Planktothrix* filaments. Cell culture flasks (600 mL polystyrene with 0.22 μm hydrophobic PP vented cap; VWR International™, Radnor, PA, USA) were kept under non-axenic conditions at 21°C, applying 10.9 μmol m^2^ s^−1^ PAR and a 16:8 light:dark cycle in AquaLytic incubators (180 L, Liebherr, Germany). *D. magna* was grown in pre-filtered (0.7 μm GF/F) lake water from Lake Lunz diluted with 10 V/V % of ADaM medium (Klüttgen *et al.*, 1994). *Daphnia* were fed with 50% *Scenedesmus* sp. and 50% *Chlamydomonas* sp. >1 mg C L^−1^; both feeding cultures were also grown in WC medium.

### ^13^C-labeling of diet sources

*Planktothrix* and chytrid-infected *Planktothrix* diet cultures were labeled with ^13^C using NaH^13^CO_3_ (98 atom%, Sigma-Aldrich, USA) in the WC medium. Before each feeding occasion, diet cultures were diluted (1:1 v/v) with either non-labeled or labeled WC medium using SIMAX 2000 mL glass bottles filled up completely to avoid air space under the caps and kept under the culturing conditions for 24 h. We did not manipulate water temperature for the diets, which would have modified their ^13^C values and made it thus impossible to compare *Daphnia* response among the treatments to diet *versus* heat. We expressed the ^13^C uptake efficiency based on all the pairwise differences in δ^13^C_FA_ between the labeled and the non-labeled replicates for each FA (i.e. Δ^13^C_FA_ = δ^13^C_FA-labeled_-δ^13^C_non-labeled_).

### Experimental design

The two diet treatments were: (1) *Planktothrix* (“P”) and (2) chytrid-infected *Planktothrix* (“PC+”), containing also free-swimming chytrid zoospores (“Z”). Two water temperature treatments were applied: (1) keeping the experimental bottles at 18°C (“T-”; temperature set in the experimental climate room), or (2) in a 24 ± 1°C water bath (“T+”) using an aquarium heater (AniOne, 25 W, MultiFit Tiernahrung GmbH, Germany). The water temperature in the experimental bottles (Corning^®^ International, 1 L square polycarbonate storage flasks) varied by ±0.5°C during the entire experiment. The *Daphnia* were either fed with non-labeled or with ^13^C-labeled cultures. All treatments—applying a full factorial design—were performed in triplicates, resulting in 24 experimental units.

At the start of the experiment, bottles contained 30 *Daphnia* neonates that were <48 h old. The volume was constantly kept at 1 L, and the bottles were covered with a non-transparent plastic plate in a non-hermetic way. A 16:8 light:dark cycle with 1.4 μmol m^2^ s^−1^ PAR during the day-light phase was applied. We replaced the medium and provided new diets to *Daphnia* every other day, corresponding to 1 ± 0.01 mg C L^−1^ in all treatments. First, the cultures’ carbon content was analyzed prior to the experiment (Abonyi *et al.*, 2023). Subsequently, we approximated the carbon content based on dry weight per volume for each feeding occasion (i.e. adding different diet volumes to meet the 1 mg C L^−1^). *Daphnia* were observed daily for survival and egg production. Individuals lying at the bottom of the bottles with no movement for 5 min were considered dead and removed. To reliably compare the PUFA content of *Daphnia* across treatments, ambient and heat treatments were stopped separately at the onset of the first successful hatching.

### Life history of *Daphnia*

We quantified *Daphnia* survival as the % of surviving individuals. The somatic growth rate (GR) of the animals was calculated as

$\mathrm{GR}=\left(\ln \left({\mathrm{DW}}_{\mathrm{end}}\right)-\ln \left({\mathrm{DW}}_{\mathrm{start}}\right)\right)/\mathrm{d},\mathrm{where}$

*DW_end_* was the average individual dry weight in each experimental bottle at the end of the experiment,

*DW_start_* was the average individual dry weight of *Daphnia* neonates at the start of the experiment (average of 3 × 20 individuals)

and *d* was the duration of the experiment in days. We quantified the number of eggs produced by *Daphnia* (ind^−1^) and egg production rates calculated from the total number of eggs produced over time (day^−1^).

### Elemental C, N, P compositions of diet and *Daphnia*

The elemental C, N and P contents of diets and chytrid zoospores were analyzed in triplicates at the beginning of the experiment (Day 1), every second time of feeding (Day 4, Day 8, Day 12) and right after the experiment (*n* = 15). *Daphnia* were analyzed for C, N and P in triplicates at the start (T_0_) and the end of the experiment (T_E_). We separated chytrid zoospores from *Planktothrix* filaments by filtering ~100 mL culture five times through a 20 μm mesh, followed by a single filtration through a 5 μm mesh. The absence of *Planktothrix* in chytrid zoospore samples was evaluated using a Nikon Eclipse TS100 inverted microscope at 200× magnification. Diets were collected on muffled and pre-weighed GF/F Whatman™ filters, dried for 48 h, weighed and folded in tin capsules. *Daphnia* were frozen (−80°C), then freeze-dried for 48 h (VirTis benchtopK, VWR International), weighed and put in tin capsules. C and N were measured using an elemental analyzer (Flash 2000, Thermo Fisher Scientific International; Waltham, MA, USA), linked to a mass spectrometer (Delta V Advantage, Thermo Fisher Scientific International; for more details, *see*  Abonyi *et al.*, 2023). Using the USGS standards, the *δ*^13^C values were referenced to Vienna PeeDee Belemite (^13^C:^12^C = 0.01118). P was measured as particulate organic phosphorus based on the ascorbic acid colourimetric method following persulfate digestion (APHA, 1998). Elemental C:N, C:P and N:P ratios were expressed as molar ratios.

### Fatty acids and compound-specific carbon isotope analyses

The fatty acids (FA) and their compound-specific carbon isotopes (i.e. CSI data on *δ*^13^C_FA_) were analyzed in the diets (i.e. *Planktothrix*, chytrid-infected *Planktothrix* excluding chytrid zoospores, chytrid zoospores) and *Daphnia*. Individual diet sources were collected on muffled and pre-weighed GF/F Whatman™ filters (47 mm, 0.7 μm pore size), stored at −80°C then freeze-dried (VirTis benchtopK, VWR International). *Daphnia* were also freeze-dried and weighted ~1 mg dry-weight into tin caps. Lipids were extracted from the freeze-dried samples using a mix of chloroform-methanol (2:1), following the protocol described in Heissenberger *et al.* (2010). Fatty acids were derivatized to methyl esters by incubation with 1% H_2_SO_4_ in methanol at 50°C for 16 h. Fatty acid methyl esters (FAME) were dried under N_2_ and dissolved in hexane. For quantification via flame ionization, FAME were separated using a GC (Trace™ 1 310, Thermo Fisher Scientific International) equipped with a Supelco™ SP-2560 column (100 m × 0.25 mm × 0.2 μm). The analytical setup allowed us to separate cis and trans isomers and components with different double bond positions. We, however, did not report trans-FA as they were scarce, if at all present. ^13^C CSI analysis was performed using a GC (Trace™ 1 310, Thermo Fisher Scientific International), connected via a ConFlo IV (Thermo Fisher Scientific International) to an Isotope Ratio Mass Spectrometer (IRMS, DELTA V Advantage, Thermo Fisher Scientific International). Samples were run against certified Me-C20:0 standards (USGS70: δ^13^C = −30.53‰, USGS71: δ^13^C = −10.5‰ and USGS72: δ^13^C = −1.54‰), which were used for drift and linear correction (for more details, see Abonyi *et al.*, 2023).

### Quantitative PUFA retention by *Daphnia*

We compared the retention of LIN, GLA, ARA, ALA, SDA and EPA by *Daphnia* among treatments in two complementary ways: first, by comparing FA mass fractions per unit biomass (i.e. μg FA mg^−1^), and, second, based on the pairwise difference in δ^13^C_FA_ values between the labeled and the non-labeled replicates for each FA (i.e. Δ^13^C_FA_ = δ^13^C_FA-labeled_-δ^13^C_non-labeled_). The higher the value, the more efficient the PUFA retention, including dietary uptake and endogenous bioconversion sources. Furthermore, for comparing the efficiency of the endogenous conversion of short-chain (LIN, ALA) with long-chain PUFA (ARA, EPA) by *Daphnia*, we expressed the ratios of FA retentions between the pairs, i.e. Δ^13^C_ARA_/Δ^13^C_LIN_ and Δ^13^C_EPA_/Δ^13^C_ALA_. The higher the value, the more efficient the endogenous FA conversion.

### Statistical analyses

We visualized *Daphnia* survival (%) and egg production (eggs ind^−1^) by fitting linear (LM) and non-linear (GAM; Wood 2011, 2017) regressions to time trends. We selected the best model based on Akaike’s information criterion (AIC). We tested for significant differences in fitness parameters (i.e. survival, somatic growth rates, eggs.ind^−1^, egg production rate) and PUFA retention efficiencies among treatments using Kruskal–Wallis rank sum tests (Hollander and Wolfe, 1973). In case of significant differences among treatments, we ran a pairwise comparison using the Wilcoxon rank sum test (Bauer, 1972). We controlled *P*-values for multiple testing by applying the false discovery rate approach (Benjamini and Hochberg, 1995). We tested for significant differences in FA content between two groups (ambient and heat) using the Wilcoxon rank sum test. We compared heat *versus* diet effects by calculating Wilcoxon effect sizes with the wilcox_effsize() function in the *rstatix* R package (Kassambara, 2023). The FA data were ln(x + 1) transformed for data visualization and statistical analyses. The level of statistical significance was set at *p* < 0.05 in all cases. All data analyses and visualizations were performed in R with RStudio Version 1.1.383 (R Core Team, 2019).

## RESULTS

### *Daphnia* fitness

**Fig. 1:**
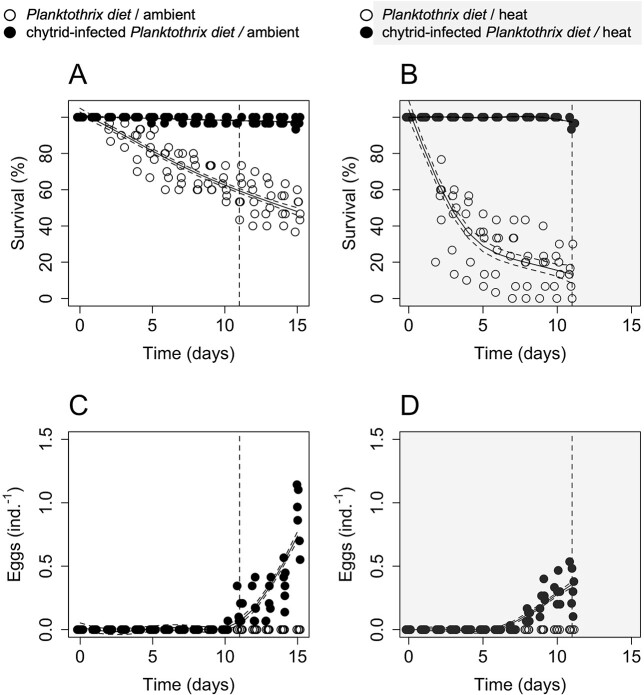
(**A**) *Daphnia* survival (% from 30 individuals per bottle) at ambient water temperature (18°C); and (**B**) *Daphnia* survival in the heat water temperature treatment (24°C); (**C**) the number of eggs produced per *Daphnia* at 18°C; and (**D**) the number of eggs produced per *Daphnia* at 24°C. The empty circles are *Daphnia* feeding on *Planktothrix*; the full circles represent *Daphnia* feeding on chytrid-infected *Planktothrix.* The vertical dashed lines indicate Day 11, the onset of the first successful reproduction of *Daphnia* feeding on the chytrid-infected diet under heat (i.e. when the heat treatment was stopped). Trend lines are based on non-linear regression analyses (GAM), except a linear regression (LM) for the chytrid-infected diet on (A).

*Daphnia* feeding on the chytrid-infected diet under heat reproduced successfully on Day 11, i.e. the end of the heat treatment. *Daphnia* on the ambient water temperature reproduced on Day 15, i.e. the end of the ambient water temperature treatment. *Daphnia* feeding on the sole *Planktothrix* diet did not develop eggs during the entire experiment, and they started to die off from Day 2 (Fig. 1A,B). The decline in survival was non-linear under both the heat and ambient water temperatures treatments (GAM, *p* < 0.001, in both cases). The survival decline was more pronounced with heat, indicated by a significantly lower survival under heat already at Day 2 (pairwise Wilcoxon, *p* < 0.01; *r* > 0.8 for heat effect).

Survival (%) differed significantly among the treatments at Day 11 (S1A, Kruskal–Wallis, chi-squared = 20.405, df = 3, *p* < 0.001). Heat significantly reduced survival only in *Daphnia* feeding on the sole *Planktothrix* diet (ambient: 58.9 ± 10.9% (mean ± SD), heat: 13.3 ± 10.1%; pairwise Wilcoxon, *p* < 0.01), while survival of *Daphnia* feeding on chytrid-infected diet remained unaffected by heat (ambient: 97.8 ± 1.7%, heat: 96.7 ± 2.1%; pairwise Wilcoxon, *p* > 0.05). The heat effect on *Daphnia* survival was large if animals were feeding on the sole *Planktothrix* diet (*r* > 0.8) and small in the case of the chytrid-infected diet (*r* < 0.3).

Somatic growth rates differed significantly among the treatments (S1B, Kruskal–Wallis, chi-squared = 17.561, df = 3, *p* < 0.001). It remained unaffected by heat (pairwise Wilcoxon, *p* > 0.05), but it was significantly enhanced by chytrid-infected diet (*p* < 0.01). Effect sizes indicated strong diet effects (*r* > 0.8) but small heat effects (*r* < 0.3) on *Daphnia* growth rates.

*Daphnia* feeding on the chytrid-infected diet started to produce eggs on Day 7 under heat, while at the ambient water temperature on Day 10 (Fig. 1C,D). Heat significantly reduced the number of eggs produced per *Daphnia* when it was compared at the day of the first successful reproduction (Day 11 under heat: 0.33 ± 0.16 eggs.ind-1 (mean ± SD), Day 15 under the ambient condition: 0.89 ± 0.23 eggs.ind^−1^, Wilcoxon, *W* = 36, *p* < 0.01). Egg production rate differed significantly among the treatments (S1C, Kruskal–Wallis, chi-squared = 20.341, df = 3, *p* < 0.001). It significantly increased in *Daphnia* feeding on chytrid-infected *Planktothrix* (pairwise Wilcoxon, *p* < 0.01), which positive effect was significantly reduced by heat (pairwise Wilcoxon, *p* < 0.01). The diet effect on the *Daphnia* egg production rate was large (*r* > 0.8), as well as the heat effect reducing the egg production rates of animals feeding on the chytrid-infected diet (*r* = 0.693).

### Individual PUFA transfer to *Daphnia*

**Fig. 2:**
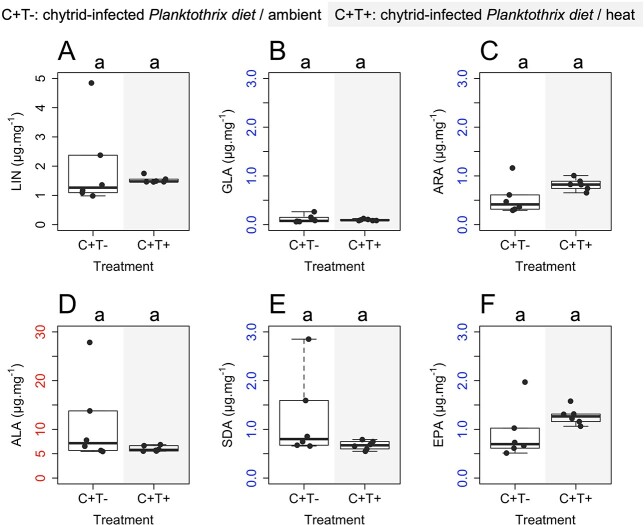
PUFA content of *Daphnia* feeding on the chytrid-infected cyanobacterium *Planktothrix* and chytrid zoospores (C+) at the time of the first successful reproduction. T- stands for ambient water temperature, while T+ stands for heat. (**A**) linoleic acid (18:2n-6, LIN); (**B**) gamma-linoleic acid (18:3n-6, GLA); (**C**) arachidonic acid (20:4n-6, ARA); (**D**) alpha-linolenic acid (18:3n-3, ALA); (**E**) stearidonic acid (18:4n-3, SDA); (**F**) eicosapentaenoic acid (20:5n-3, EPA). Different ranges of the y-axes are color-coded. The letters indicate significant differences between water temperature treatments based on Wilcoxon rank sum tests (*n* = 6 in all cases; different letters indicate significant differences).

Due to the high mortality of *Daphnia* feeding on the sole *Planktothrix* diet, it was only possible to analyze the PUFA content in *Daphnia* feeding on the chytrid-infected diet. The individual PUFA contents in *Daphnia* feeding on chytrid-infected *Planktothrix* did not differ significantly between the ambient and heat treatments (Wilcoxon rank sum tests, *p* > 0.05 in all cases, Fig. 2). ALA had the highest mass fractions of the identified short-chain PUFA in *Daphnia* (ambient: 11.2 ± 8.7 μg mg^−1^ (mean ± SD), heat: 6.0 ± 0.6 μg mg^−1^) followed by LIN (ambient: 2.0 ± 1.5 μg mg^−1^, heat: 1.5 ± 0.1 μg mg^−1^) and SDA (ambient: 1.2 ± 0.9 μg mg^−1^, heat: 0.7 ± 0.1 μg mg^−1^) and GLA (ambient: 0.1 ± 0.08 μg mg^−1^, heat: 0.1 ± 0.02 μg mg^−1^). EPA had the highest mass fractions of the identified long-chain PUFA in *Daphnia* (ambient: 0.9 ± 0.5 μg mg^−1^, heat: 1.3 ± 0.2 μg mg^−1^), followed by ARA (ambient: 0.5 ± 0.3 μg mg^−1^, heat: 0.8 ± 0.1 μg mg^−1^) and DHA (ambient: 0.1 ± 0.07 μg mg^−1^, heat: 0.05 ± 0.01 μg mg^−1^).

### ^13^C-labeling of dietary PUFA and their retention by *Daphnia* based on CSI analysis

**Fig. 3:**
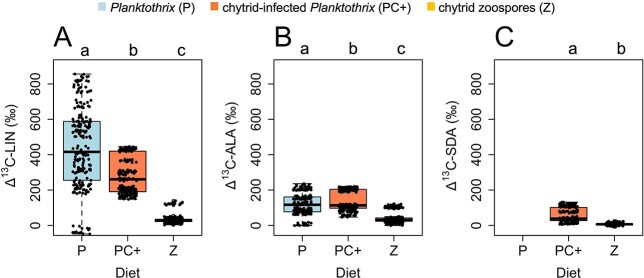
^13^C uptake via key PUFA in the diet sources based on all pairwise differences between labeled and non-labeled FA (*n* = 225 for each pair) (**A**) linoleic acid (18:2n-6, LIN); (**B**) alpha-linolenic acid (18:3n-3, ALA); (**C**) stearidonic acid (18:4n-3, SDA). The letters stand for significant differences in ^13^C uptake via key PUFA among diet sources based on Pairwise Wilcoxon rank sum tests.

The ^13^C uptake via LIN, ALA and SDA differed significantly among diet sources (Kruskal–Wallis rank sum tests, chi-squared_LIN_ = 415.27 (df = 2), chi-squared_ALA_ =  277.47 (df = 2), chi-squared_SDA_ = 295.48 (df = 1), *p* < 0.001 in all cases). ^13^C uptake via LIN was the highest in *Planktothrix* (438 ± 229‰), significantly lower in chytrid-infected *Planktothrix* (284 ± 104‰), and again, significantly lower in chytrid zoospores (34 ± 26‰)(Fig. 3A). ^13^C labeling of ALA was the highest in chytrid-infected *Planktothrix* (139 ± 57‰), significantly lower in the cyanobacterium diet (121 ± 60‰), and chytrid zoospores (43 ± 33‰)(Fig. 3B). SDA was absent in *Planktothrix* but present in the chytrid-infected cyanobacterium and the chytrid zoospores (Fig. 3C). ^13^C uptake via SDA was significantly higher in chytrid-infected *Planktothrix* (56 ± 39‰) than in the chytrid zoospores (6 ± 5‰). Diet sources did not contain ARA or APA (see also S3A).

**Fig. 4:**
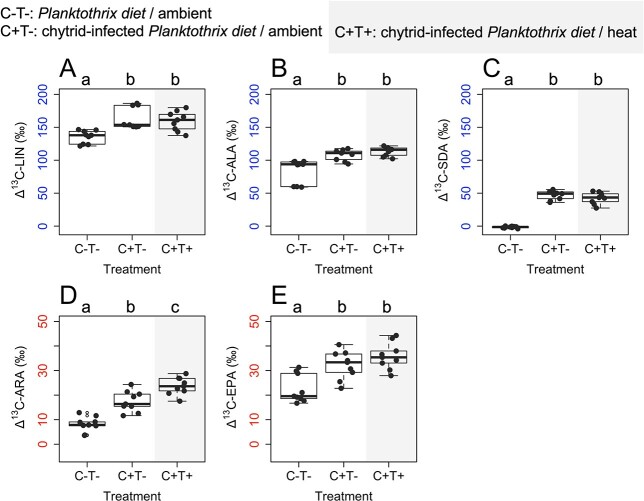
^13^C retention via key PUFA in *Daphnia* at the onset of the first successful reproduction among treatments (all pairwise differences between labeled and non-labeled FA, *n* = 9 for each pair) (**A**) linoleic acid (18:2n-6, LIN); (**B**) alpha-linolenic acid (18:3n-3, ALA); (**C**) stearidonic acid (18:4n-3, SDA); (**D**) arachidonic acid (20:4n-6, ARA); and (**E**) eicosapentaenoic acid (20:5n-3, EPA). The letters indicate significant differences in FA retention of *Daphnia* among treatments based on Pairwise Wilcoxon rank sum tests.

Due to the high mortality of *Daphnia* feeding only on *Planktothrix* under heat, *Daphnia* from this treatment could not be analyzed. Among the other treatments, ^13^C retention in *Daphnia* significantly differed via LIN, ALA, SDA, ARA and EPA (Fig. 4; Kruskal–Wallis rank sum tests, p_LIN_ < 0.01, p_ALA_ < 0.001, p_SDA_ < 0.001, p_ARA_ < 0.001, p_EPA_ < 0.01). At ambient water temperature, ^13^C-labeling indicated significantly enhanced LIN, ALA, SDA, ARA and EPA retention in *Daphnia* feeding on the chytrid-infected *Planktothrix* diet (Pairwise Wilcoxon rank sum tests, p_LIN_ < 0.001, p_ALA_ < 0.01, p_SDA_ < 0.001, p_ARA_ < 0.001, p_EPA_ < 0.05). The heat treatment did not affect PUFA retention significantly in *Daphnia* feeding on the chytrid-infected diet (Pairwise Wilcoxon rank sum tests, *p* > 0.05, in all cases), except for ARA, in which case heat significantly enhanced the retention (Fig. 4D, Pairwise Wilcoxon rank sum tests, *p* < 0.01). Effect sizes indicated large effects of the diet type on the retention of individual FA (*r* > 0.6 in all cases), while heat only affected ARA retention largely (*r* > 0.6). In all other cases, heat only had a small effect on the retention of individual FA (*r* < 0.3).

**Fig. 5:**
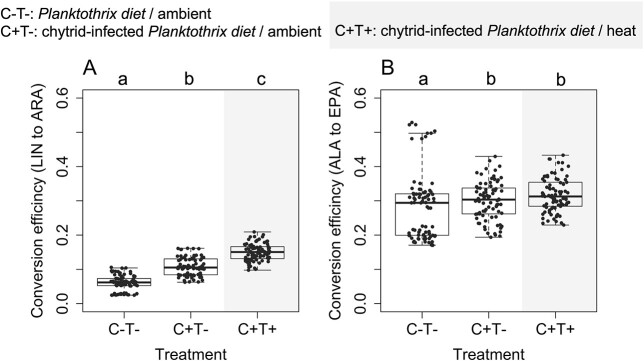
Efficiency in the endogenous bioconversion of (**A**) LIN to ARA (n-6 chain); and (**B**) ALA to EPA (n-3 chain) in *Daphnia* at the onset of the first successful reproduction among treatments (boxplots show all pairwise ratios between Δ^13^C_ARA_/Δ^13^C_LIN_ and Δ^13^C_EPA_/Δ^13^C_ALA_; *n* = 81, for each treatment). The letters indicate significant differences in FA conversion efficiencies of *Daphnia* among treatments based on Pairwise Wilcoxon rank sum tests.

Endogenous conversion of short-chain to long-chain PUFA along with the n-3 chain (i.e. ALA to EPA) occurred with a ~3x higher conversion efficiency than the n-6 chain (i.e. LIN to ARA, Fig. 5). The chytrid-infected diet combined with heat significantly increased the LIN to ARA conversion (Fig. 5A, Pairwise Wilcoxon rank sum tests, *p* < 0.001, in all cases). Chytrid-infected diet had a large impact on LIN to ARA conversion (*r* > 0.7), which was also largely impacted by heat (*r* > 0.8). Along with the ALA to EPA chain, the chytrid diet alone slightly but significantly (*p* < 0.05), and the chytrid diet together with the heat highly significantly (*p* < 0.001) enhanced the endogenous conversion (Fig. 5B, Pairwise Wilcoxon rank sum tests). Effect sizes, however, indicated only small effects of chytrids and heat on the ALA to EPA conversion efficiency (*r* < 0.3 in all cases).

## DISCUSSION

The high mortality of *Daphnia* neonates exposed to heat after 2 days confirmed our hypothesis that a +6°C water temperature increase would harm the zooplankton consumer feeding on the sole *Planktothrix* diet. Our data also confirmed that the chytrid-infected diet could support *Daphnia* fitness under stress conditions induced by the combined impact of heat and limiting diet (i.e. organic matter quantity, nutritional quality). Chytrids could enhance the dietary supply and subsequent retention of key PUFA by *Daphnia* at both water temperature treatments. Our study thus provides the first experimental evidence for the positive diet quality effect of chytrids in terms of PUFA as a possible key mechanism providing insurance against the detrimental effect of heat under cyanobacteria blooms in nature.

### PUFA transfer in the mycoloop under heat

Chytrids can perform trophic upgrading along with the n-3 and n-6 PUFA chains (Rasconi *et al.*, 2020), resulting in enhanced PUFA retention in *Daphnia* (Abonyi *et al.*, 2023). Here we show that enhanced PUFA retention by *Daphnia* with the chytrid-infected diet remains largely unaffected by a +6°C water temperature increase if the diet conditions are limiting in terms of both organic matter quantity and nutritional quality. Due to the lack of ARA and EPA in the diet sources and the quantitative limitation in diet, LIN and ALA are converted into ARA and EPA by the *Daphnia*, respectively (Bec *et al.*, 2003; Abonyi *et al.*, 2023). The compound-specific fatty acid data clearly show that the endogenous bioconversion of ALA to EPA is more efficient than the conversion of LIN to ARA in *Daphnia*. While the conversion rate is low and the EPA content produced may still limit somatic growth (Ilić *et al.*, 2021 and herein references), here we see that it is strongly enhanced by chytrids, providing a plausible mechanistic link between chytrid-mediated enhanced n-3 PUFA retention and *Daphnia* fitness. The n-3 conversion pathway producing EPA, a critical functional LC-FA for growth and reproduction (Becker and Boersma, 2003), remains unaffected by heat if chytrids are present in the diet. The sustained retention under heat also holds for the n-3 PUFA SDA, as yet, however, with unknown energetic benefits (Abonyi *et al.*, 2023).

Surprisingly, while the chytrid diet enhances n-3 over n-6 PUFA retention (i.e. in response to enhanced endogenous conversion), the heat significantly enhances the n-6 PUFA ARA retention via a more efficient LIN to ARA conversion. While this may suggest a crucial physiological role for ARA, the diet quality effect of chytrids in the conversion was weak. Thus, its potential benefits against heat conditions remain to be elucidated. The more efficient endogenous n-3 PUFA conversion and more stable EPA content in *Daphnia* may show that EPA is the limiting factor over ARA. Accordingly, as soon as a certain amount of EPA is acquired, *Daphnia* may reduce its conversion rate and start reproducing (Pilecky *et al.*, 2021). This might be supported by the fact that *D. magna* preferentially store EPA, which appears in higher mass fractions than ARA in the eggs (Becker and Boersma, 2005). If this is the case, high conversion rates from LIN to ARA might be an unnecessary by-product—process for the animal; in case of its high need, ARA levels would also remain more stable (Pilecky *et al.*, 2021).

On dietary PUFA, however, a recent study suggested that *Daphnia* could be even more susceptible to the lack of ARA than EPA (Ilić *et al.*, 2021). As both ARA and EPA were lacking in diets, our results highlighting a preferential n-3 PUFA over n-6 PUFA conversion may not support the view of Ilić *et al.* (2021). Moreover, the potential effect of the chytrid-synthesized SDA on the endogenous conversion pathway of ALA to SDA remains unknown. The preferential n-3 PUFA conversion by the *Daphnia* might have also been enhanced by the favored n-3 PUFA upgrading of chytrids (Rasconi *et al.*, 2020; Abonyi *et al.*, 2023), which would provide a mechanistic link between increased dietary quality for *Daphnia* and the enhanced performance of the animals.

### The chytrid-mediated PUFA insurance against the heat

Ectothermic organisms can genetically adapt to higher temperatures (Geerts *et al.*, 2015). Also, the physiological plasticity of animals allows changes in biochemical characteristics to counter the negative impact of heat. For example, *Daphnia* adjust its FA content with decreasing PUFA toward higher temperatures (Zeis *et al.*, 2019). Masclaux *et al.* (2009) argued that the unsaturation level of fatty acids increases toward lower temperature in membrane lipids, which makes the animal more susceptible to heat-induced oxidative stress (Zeis *et al.*, 2019). Here we show that *Daphnia* acclimated at 18°C for >1 year can successfully cope with a sudden +6°C water temperature increase and counteract energy and PUFA limitation by *Planktothrix* if chytrids are also available in the diet.

Our results suggest that trophic upgrading within the mycoloop is a crucial and strong mechanism supporting *Daphnia* fitness under a significant water temperature increase, e.g. during heatwaves. Extreme temperatures may amplify the deleterious effect of warming on the performance and fitness of ectothermic organisms (Kingsolver *et al.*, 2013), especially under reduced diet availability (Huey and Kingsolver, 2019). This certainly holds for late summer phytoplankton in the temperate region, where large-sized, hardly palatable phytoplankton often dominate under eutrophic conditions (Sommer *et al.*, 1986; Ptacnik *et al.*, 2008; O’Neil *et al.*, 2012).

Although our data suggest a positive diet quality effect of trophic upgrading by chytrids between *Planktothrix* and *Daphnia*, cyanobacteria lack growth-supporting sterols (Martin-Creuzburg *et al.*, 2010). Thus, trophic upgrading is limited to somatic effects of fatty acids for *Daphnia* and, importantly, subsequent consumers. Since we lack experimental evidence for the combined diet quality effect of sterol and PUFA-upgrading at the phytoplankton–zooplankton interface, including chytrids, we speculate that trophic upgrading by chytrids remains a valid mechanism, irrespective of the type of algae (Rasconi *et al.*, 2020). The relative importance of sterol- *versus* PUFA-upgrading and different host types in supporting *Daphnia* performance under heat remains to be elucidated.

The elemental composition of phytoplankton can also constrain zooplankton fitness under low phosphorus concentrations (Elser *et al.*, 2001). *Daphnia* feeding on chytrids, however, remained unchanged in their C:P ratios, irrespective of water temperature, even though the chytrid zoospores contained significantly more phosphorus than *Planktothrix* and the chytrid-infected *Planktothrix* (Fig. S2). Instead, lipids and their FA, such as EPA and ARA, were the key molecules that improved *Daphnia* fitness (Müller-Navarra *et al.*, 2000; Brett *et al.*, 2009). While Daphnia can adapt EPA to higher water temperatures to sustain membrane fluidity if the diet is neither limiting in quantity nor quality (Schlechtriem *et al.*, 2006), they may function differently in limiting diet conditions of organic matter quantity and nutritional quality. We argue that *Daphnia* preferentially convert along with the n-3 chain (ALA to EPA) to sustain first survival, then growth and reproduction, which remains largely unaffected by a +6°C water temperature increase.

While zooplankton fitness decreases in response to the dietary lack of ARA and EPA (Gulati and Demott, 1997), here we show that chytrids can support *Daphnia* fitness via sustained endogenous EPA and ARA conversion, even under heat. Global warming further intensifies harmful, largely inedible PUFA-deprived phytoplankton blooms (Paerl and Huisman, 2008; Wagner and Adrian, 2009). Thus, the alternative energy and PUFA pathway via chytrid parasites may become increasingly crucial to ensure pelagic food web functioning in the future.

### The strength of diet quality *versus* heat effects

The positive effect of the chytrid diet on *Daphnia* fitness was systematically strong. In contrast, the negative heat effect was systematically small, except for large heat effects if the animals were feeding on the sole cyanobacterium diet. This emphasizes that chytrids provide a strong insurance for sustained dietary energy transfer toward higher trophic levels and can efficiently buffer PUFA requirements of *Daphnia* under heat.

## CONCLUSIONS

The presence of chytrids in an otherwise deleterious diet may strongly improve growth and reproduction in the keystone herbivore, *Daphnia*. The positive effect of chytrids on *Daphnia* growth and reproduction was particularly strong in heated conditions, underling the potential relevance of such effects under global warming. Chytrids support and improve PUFA accrual in *Daphnia* via increased dietary access to the n-3 PUFA SDA (preferential n-3 upgrading by the chytrids), which co-occurs with higher n-3 (ALA to EPA) than n-6 (LIN to ARA) PUFA upgrading by the animals. Though both were significant, chytrids' positive diet quality effect on the n-3 PUFA conversion was large but small on the n-6 PUFA conversion path. We observed that the chytrid-synthesized SDA, retained selectively by *Daphnia*, remained quantitatively unchanged with heat. Heat significantly enhanced LIN to ARA bioconversion by the consumer, but the effect was small, with unknown benefits for the consumer. We conclude that the positive dietary effect of chytrids in terms of PUFA is crucial for *Daphnia* fitness under heat and long-chain PUFA-deprived cyanobacteria blooms. Thus, chytrids likely help function pelagic food webs by improving PUFA availability for higher trophic levels under global warming.

## Supplementary Material

JPR_R2_Supplement_fin_fbad012

## Data Availability

The data of this study are available from the corresponding author upon request.
