# Supplementary material for: Chytrids alleviate the harmful effect of heat and cyanobacteria diet on Daphnia via PUFA-upgrading
Source: J Plankton Res. 2023 Apr 21;45(3):454–66. doi: 10.1093/plankt/fbad012 (PMC10243842; doi:10.1093/plankt/fbad012)
Supplement: JPR_R2_Supplement_fin_fbad012 [file jpr_r2_supplement_fin_fbad012.docx]

Supplementary material (4 pages) for

**Chytrids alleviate the harmful effect of heat and cyanobacteria diet on *Daphnia* via PUFA-upgrading**

Running head: Chytrids support *Daphnia* with PUFA under heat

^1,4,+^András Abonyi, [abonyi.andras@ecolres.hu](mailto:abonyi.andras@ecolres.hu), ORCID: 0000-0003-0593-5932

^1,3^Matthias Pilecky, [matthias.pilecky@donau-uni.ac.at](mailto:matthias.pilecky@donau-uni.ac.at), ORCID: 0000-0002-3404-5923

^1,2^Serena Rasconi, [serena.rasconi@inrae.fr](mailto:serena.rasconi@inrae.fr), ORCID: 0000-0001-6667-8904

^1^Robert Ptacnik, [robert.ptacnik@wcl.ac.at](mailto:robert.ptacnik@wcl.ac.at), ORCID: 0000-0001-7176-7653

^1,3^Martin J. Kainz, [martin.kainz@donau-uni.ac.at](mailto:martin.kainz@donau-uni.ac.at), ORCID: 0000-0002-2388-1504

1 WasserCluster Lunz – Biologische Station GmbH, Dr. Carl Kupelwieser Promenade 5, A-3293 Lunz am See, Austria

2 Université Savoie Mont Blanc, INRAE, CARRTEL, 74200 Thonon-les-Bains, France

3 Donau-Universität Krems, Dr. Karl Dorrek Straße 30, A-3500 Krems, Austria

4 Institute of Aquatic Ecology, Centre for Ecological Research, Karolina út 29., H-1113 Budapest, Hungary

+ Corresponding author: Andras Abonyi, [abonyi.andras@ecolres.hu](mailto:abonyi.andras@ecolres.hu)

*Survival, growth, and egg production rates of* Daphnia

**S1** Boxplot of *Daphnia* **(A)** survival (% from 30 individuals per bottle) at day 11 (end of the heat treatment); **(B)** somatic growth rate, and: **(C)** egg production rate among treatments. Letters stand for significant differences based on pairwise comparisons of treatment groups based on Wilcoxon rank sum test (n=6 in all cases).

*Elemental composition of diets and* Daphnia

Carbon (C) and nitrogen (N) contents did not differ significantly among diets (Kruskal Wallis, df=2, p>0.05 in both cases). Phosphorus (P) differed significantly among diet sources (Kruskal-Wallis, chi-squared = 35.966, df = 2, p<0.001). It was significantly higher in chytrid zoospores compared with both *Planktothrix* and chytrid-infected *Planktothrix* (Pairwise Wilcoxon rank sum tests, p<0.001 in both cases). P in Planktothrix and chytrid-infected Planktothrix, however, did not differ significantly (Pairwise Wilcoxon rank sum test, p>0.05). The C:N ratio (Kruskal-Wallis, chi-squared = 22.92, df = 2, p<0.001) and the C:P ratio significantly differed among diet treatments (Kruskal-Wallis, chi-squared = 33.479, df = 2, p<0.001).

Chytrid zoospores were significantly lower in C:N (p_P-Z_<0.001, p_PC+-Z_<0.01) and C:P (p_P-Z_<0.001, p_PC+-Z_<0.001) compared with the other diet sources (S2A,B; Pairwise Wilcoxon rank sum test). Pairwise Wilcoxon rank sum test). The C:P ratio was also significantly lower in the chytrid zoospores compared with the cyanobacterium and the chytrid-infected cyanobacterium (p<0.001 in both cases; Supplement Fig S2B, Pairwise Wilcoxon rank sum test).


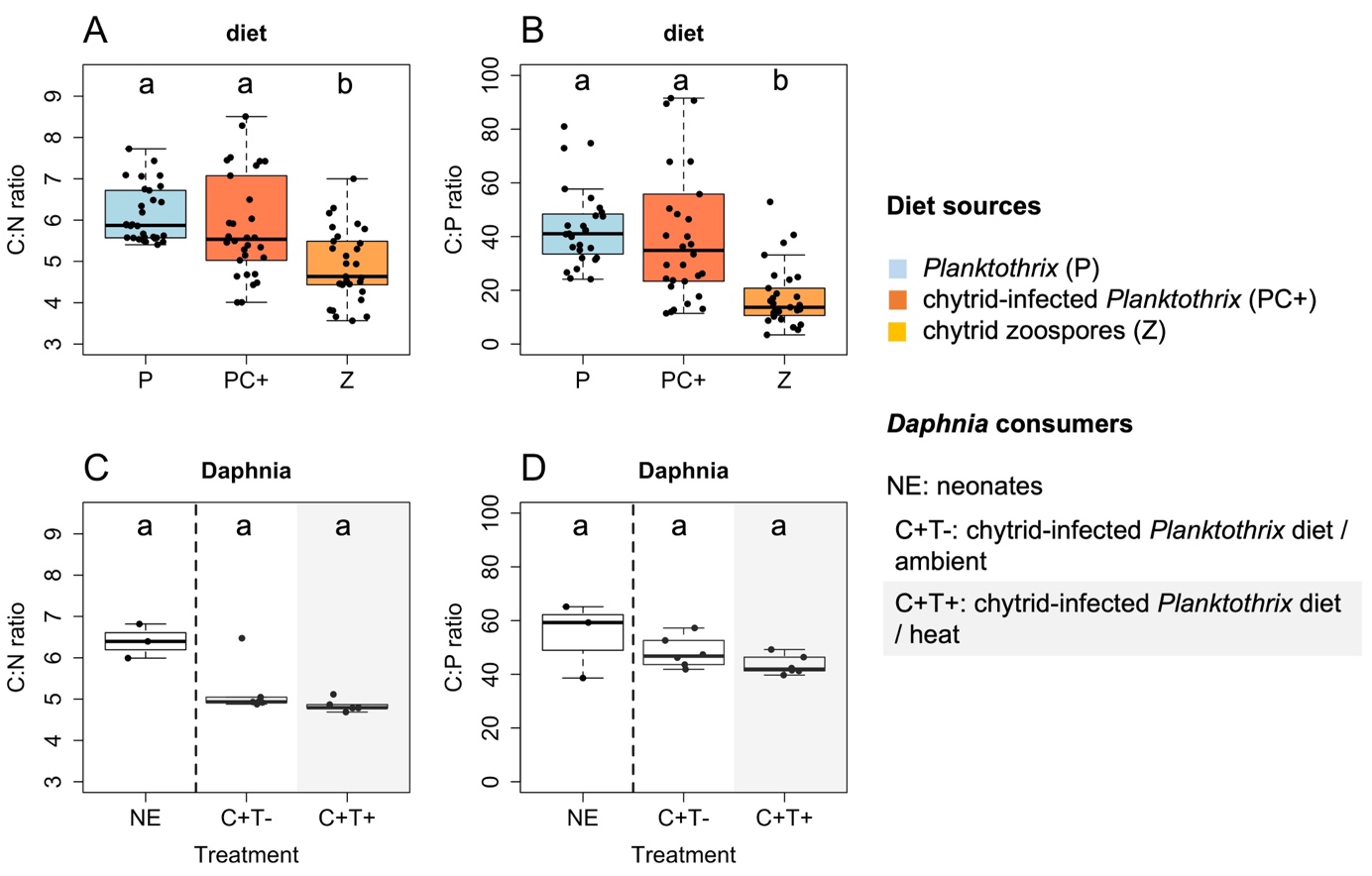


**S2** Boxplots of atomic **(A)** C:N and **(B)** C:P ratios in the diets (n=30 for each diet source); and boxplots of the atomic **(C)** C:N and **(D)** C:P ratios in *Daphnia* (n=3 for *Daphnia* neonates, and n=6 for *Daphnia* at the onset of the first successful reproduction, respectively). Letters above the boxplots denote the level of significance based on pairwise comparisons using Wilcoxon rank sum test.

The C and N content of *Daphnia* differed significantly among neonates and *Daphnia* feeding on chytrid-infected (Kruskal-Wallis rank sum test, df=2, p<0.01 in both cases). *Daphnia* feeding on the chytrid-infected diet had significantly higher C and N contents at the ambient compared with the heat treatment (Pairwise Wilcoxon rank sum test, p<0.01). The P content, however, did not differ significantly among neonates and *Daphnia* under different water temperature treatments (Pairwise Wilcoxon rank sum test, p>0.05).

The C:N ratio in *Daphnia* weakly but significantly differed among treatments (Kruskal-Wallis, chi-squared = 8.6, df = 2, p<0.05). However, in the pairwise comparisons, there was no significant differences among the *Daphnia* neonates and treatments (Pairwise comparisons using Wilcoxon rank sum test, p>0.05 in all cases). The C:P ratio in *Daphnia did not* differed significantly among treatments (Kruskal-Wallis, chi-squared = 2.4167, df = 2, p>0.05)*.*

*PUFA profile of diets and* Daphnia

The key PUFA of diets were LIN (18:2n-6), ALA (18:3n-3) and SDA (18:4n-3; S3A). The *Planktothrix* and chytrid-infected *Planktothrix* did not differ in LIN and ALA (Pairwise comparisons using Wilcoxon rank sum test, p>0.05), but chytrid zoospores had significantly lower LIN and ALA compared with the cyanobacterium and its chytrid-infected cultures (Pairwise comparisons using Wilcoxon rank sum test, p<0.001 in both cases). The sole *Planktothrix* culture did not contain SDA, while the chytrid-infected *Planktothrix* and chytrid zoospores did contain SDA in similar amounts (Pairwise comparisons using Wilcoxon rank sum test , p_PC+-Z_>0.05, p_P-PC+_<0.001, p_P-Z_<0.001).

**S3 (A)** Polyunsaturated fatty acid (PUFA) content of diets (n=15); and **(B)** PUFA content of *Daphnia* (n=3 for *Daphnia* neonates, and n=6 for *Daphnia* at the onset of the first successful reproduction, respectively). Letters stand for significant differences based on pairwise comparisons of treatment groups based on Wilcoxon rank sum test.

*Daphnia* neonates and *Daphnia* feeding on chytrid-infected diet did not differ significantly in LIN, ALA and SDA, irrespective of water temperature treatments (Pairwise comparisons using Wilcoxon rank sum test, p>0.05 in all pairwise cases). *Daphnia* feeding on chytrids, however, contained significantly higher amount of ARA (20:4n-6)and EPA (20:5n-3) compared with *Daphnia* neonates, irrespective of water temperature treatments (Pairwise comparisons using Wilcoxon rank sum test, p<0.05 in all pairwise cases).
